# Birth Weight in Consecutive Pregnancies and Maternal Cardiovascular Disease Mortality Among Spontaneous and Iatrogenic Term Births: A Population-Based Cohort Study

**DOI:** 10.1093/aje/kwad075

**Published:** 2023-05-30

**Authors:** Yeneabeba Tilahun Sima, Rolv Skjaerven, Liv Grimstvedt Kvalvik, Nils-Halvdan Morken, Kari Klungsøyr, Janne Mannseth, Linn Marie Sørbye

**Keywords:** birth weight, cardiovascular disease, cardiovascular disease mortality, consecutive pregnancies, iatrogenic delivery, pregnancy, spontaneous delivery, term birth

## Abstract

Knowledge on the association between offspring birth weight and long-term risk of maternal cardiovascular disease (CVD) mortality is often based on firstborn infants without consideration of women’s consecutive births. We studied long-term CVD mortality according to offspring birth weight patterns among women with spontaneous and iatrogenic term deliveries in Norway (1967–2020). We constructed birth weight quartiles (Qs) by combining standardized birth weight with gestational age in quartiles (Q1, Q2/Q3, and Q4) for the women’s first 2 births. Mortality was estimated using Cox regression and expressed as hazard ratios (HRs) with 95% confidence intervals (CIs). Changes in offspring birth weight quartiles were associated with long-term maternal CVD mortality. Compared with women who had 2 term infants in Q2/Q3, women with a first offspring in Q2/Q3 and a second in Q1 had higher mortality risk (HR = 1.33, 95% CI: 1.18, 1.50), while risk was lower if the second offspring was in Q4 (HR = 0.78, 95% CI: 0.67, 0.91). The risk increase associated with having a first infant in Q1 was eliminated if the second offspring was in Q4 (HR = 0.99, 95% CI: 0.75, 1.31). These patterns were similar for women with iatrogenic and spontaneous deliveries. Inclusion of information from subsequent births revealed heterogeneity in maternal CVD mortality which was not captured when using only information based on the first offspring.

## Abbreviations

BMI: body mass index
CI: confidence interval
CVD: cardiovascular disease
HR: hazard ratio
ICD: *International Classification of Diseases*
MBRN: Medical Birth Registry of Norway
Q: quartile

Low infant birth weight is associated with increased risk of maternal cardiovascular disease (CVD) mortality (1). However, there are inconsistent findings regarding the association between large infants and long-term maternal mortality (1). While the lowest CVD mortality is found among women with large infants in some populations (2–5), other investigators report a higher risk of CVD mortality among women with large babies (6–8). Most of these studies include preterm births, which are known to be independently associated with long-term maternal CVD mortality (9). To our knowledge, no previous studies have focused on these relationships among term births only, which comprise the majority of all births. Furthermore, most of the published literature pertains to women’s firstborn infants (2–8, 10) without consideration of subsequent births, which could lead to biased estimates (11). Both recurrence and order of complications in subsequent pregnancies affect mortality risk (9).

**Figure 1:**
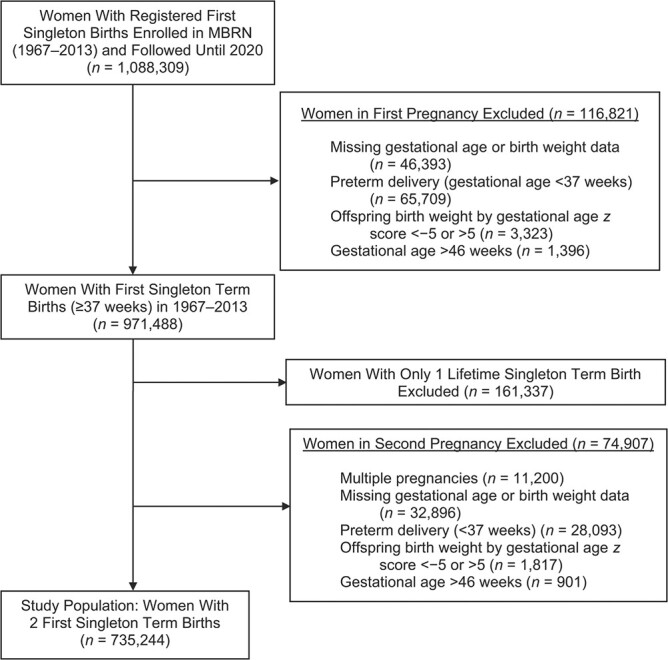
Selection of women with 2 first singleton term births from the Medical Birth Registry of Norway (MBRN) for a study of heterogeneity in maternal cardiovascular disease mortality risk according to change in offspring birth weight by gestational age, Norway, 1967–2020.

When studying the relationship between offspring birth weight and future maternal health, measures that account for birth weight variation by gestational age may be more informative than absolute birth weight (7), especially when preterm births are included (6). However, gestational age variation, even within the term period, has been shown to be related to future maternal CVD mortality (9), indicating that when studying term births only, standardizing birth weight for gestational age may also be needed. Moreover, the gradual rise in labor induction and cesarean delivery might influence both offspring gestational age and birth weight distribution (12). Except for the study by Rich-Edwards et al. (9), no study (to our knowledge) has investigated the relationship between gestational age and long-term maternal mortality specifically with regard to spontaneous and iatrogenic deliveries in term pregnancies.

In the present study, we wanted to evaluate heterogeneity in maternal CVD mortality risk according to change in offspring birth weight by gestational age among women with 2 term births. Using linked data from population-based registries in Norway, we tested the hypothesis that changes in offspring birth weight quartiles from the first pregnancy to the second influence long-term risk of maternal CVD mortality. We also wanted to evaluate whether associations differ by type of delivery (spontaneous vs. iatrogenic delivery).

## METHODS

### Data sources

This was a population-based cohort study using data from the Medical Birth Registry of Norway (MBRN), which has been based on mandatory notification of all births taking place in the country from 16 gestational weeks onward since 1967 (13). Data are collected on demographic characteristics, reproductive history, and the mother’s health before and during pregnancy, including delivery complications and infant outcomes. The attending clinician is responsible for filling out the forms. Information was based on free text descriptions until 1998, while checkboxes were added in 1999. By means of the national identification number assigned to all residents of Norway, data from the MBRN were linked to the Norwegian Cause of Death Registry and the National Education Database of Statistics Norway. Births to the same women were identified, keeping the mother as the unit of analysis.

Our study complied with the Declaration of Helsinki and was approved by the Regional Committee for Medical and Health Research Ethics. Informed consent was not required, since the data were deidentified, and the researchers did not have any contact with participants. We followed the STROBE checklist (Strengthening the Reporting of Observational Studies in Epidemiology; https://www.strobe-statement.org/) for cohort studies (see Web Table 1, available at https://doi.org/10.1093/aje/kwad075).

### Inclusion and definitions

We included women with 2 or more births whose first birth was registered between 1967 and 2013, providing women with at least 7 years to have finished their reproduction. About 95% of Norwegian women have their second child within 7 years of the first (11). We focused on women’s first 2 births, and among these we excluded women with multiple pregnancies, women who were missing data on gestational age or birth weight, preterm deliveries (<37 weeks), pregnancies with a standardized offspring birth weight (*z* score) less than −5 or greater than 5, and pregnancies with a gestational age greater than 46 weeks.

Term delivery was defined as birth at 37 weeks’ gestation or later. Estimation of gestational age was based on the date of the last menstrual period. Ultrasound-based gestational age estimation, available in the MBRN from 1999 onward, was used for women with missing information on the last menstrual period or with a difference of more than 10 days between the last menstrual period and ultrasound-based estimation. The date of embryo transfer plus 14 days was used for infants conceived by in vitro fertilization (available in the MBRN from 1985). Birth weight was registered in grams. The validity of registered gestational age and birth weight data in the MBRN is high (14). Estimates of birth weight by gestational age *z* score were calculated using mean values and standard deviations from the Norwegian population (12). We calculated parity-specific standardized quartiles (25th, 50th, and 75th percentiles) of offspring birth weight (in grams) by gestational week for women’s first and second births, respectively. The parity-specific cutoff points for offspring birth weight quartiles were defined from a population of women with singleton term births. Based on the linear trend between quartiles of birth weight by gestational age and maternal CVD mortality, we merged Q2 and Q3. Offspring birth weight quartiles for the first and second births (Q1, Q2/3, Q4) were combined into one exposure variable consisting of 9 categories: Q1-Q1, Q1-Q2/3, Q1-Q4, Q2/3-Q1, Q2/3-Q2/3 (reference category), Q2/3-Q4, Q4-Q1, Q4-Q2/3, and Q4-Q4. The changes in offspring birth weight quartiles from the first pregnancy to the second constituted the pattern of offspring birth weight by gestational age quartile.

Medical interventions that end pregnancies before their natural endpoint, such as induction of labor and prelabor cesarean delivery, might influence offspring birth weight quartiles. To assess whether our results differed among women who delivered spontaneously or had iatrogenic deliveries, we stratified the analyses on the basis of type of labor onset. “Spontaneous delivery” included women with spontaneous labor onset in both pregnancies, while women with either induced labor or prelabor cesarean delivery in the first and/or second pregnancy were grouped as having “iatrogenic delivery.”

Information on cigarette smoking (no (referent) or yes (daily/sometimes)) and body mass index (BMI; weight (kg)/height (m)^2^) was available from 1999 onwards and 2006 onwards, respectively.

Maternal mortality was registered in the Cause of Death Registry using *International Classification of Diseases* (ICD) codes. For our main analyses, we combined deaths due to ischemic heart disease (*International Classification of Diseases, Eighth Revision* (ICD-8) and *International Classification of Diseases, Ninth Revision* (ICD-9) codes 410–414; *International Classification of Diseases, Tenth Revision* (ICD-10) codes I20–I25) and cerebrovascular disease/stroke (ICD-8 and ICD-9 codes 430–438; ICD-10 codes 160–I69) into one group (“cardiovascular deaths”). We also examined all-cause mortality, circulatory system diseases (ICD-8 and ICD-9 codes 390–459; ICD-10 codes I00–I99), and noncirculatory diseases (all deaths other than those included in the “circulatory system diseases” definition) mortality.

### Statistical analyses

Frequency and contingency tables were used when constructing parity-specific cutoff points for all first and second births (Web Table 2). Categorical variables were summarized using proportions, while continuous variables were summarized with mean values and standard deviations. Mortality was estimated using Cox proportional hazards models providing hazard ratios (HRs) and 95% confidence intervals (CIs), with woman’s age as the underlying time variable. Women were considered at risk of CVD mortality from their last pregnancy to either death or censoring, whatever came first. In our data there seemed to be no excess maternal CVD mortality by pregnancy complications after the age of 70 years. As a result, we right-censored all observations at the age of 70 years (if women were not already deceased). Schoenfeld residuals were checked for any evidence of deviation from the proportional hazards assumption. In addition to the cause-specific hazard models, we also fitted a subdistribution hazard model to account for competing risk (15).

We performed 2 main analyses when estimating maternal CVD mortality risk. First, we used only information from women’s first birth. Women with spontaneous first deliveries in Q2/3 were designated the reference group. Second, we calculated mortality risks by combining standardized birth weight data from first and second births. Women with both offspring in Q2/3 and spontaneous delivery were the reference group in these analyses. Estimates were adjusted for maternal age at first birth (years; continuous), year of last delivery, maternal education (<11 years (low) vs. ≥11 years (high; referent)), and pregnancy complications.

Several sensitivity analyses were performed. We excluded women with known risk factors for CVD (in both pregnancies), including pregnancy complications (chronic/gestational hypertension, pregestational/gestational diabetes mellitus, perinatal loss (included stillbirths and early neonatal death occurring within 1 week after birth), placental abruption and preeclampsia (16), offspring congenital malformations (17), and subfertility issues (conception by in vitro fertilization) (18)). In addition, we performed separate analyses to minimize confounding by ethnicity (19) (analyzing only women of Nordic origin), to account for the potential influence of different fathers (20) (analyzing women with the same partner), to account for interpregnancy interval (categorized as <12.0 months, 12.0–23.9 months, 24.0–35.9 months, and ≥36.0 months) (21), to account for full-term gestations (restricted to 39–41 weeks) (9), and to assess the influence of higher parity on mortality patterns (analyzing the first and third offspring among the first 3 term deliveries).

Due to missing information on maternal smoking and prepregnancy BMI, we also performed E-value–based sensitivity analysis to determine the extent to which unmeasured confounding may have influenced the observed association (22). The E-value estimates the HR for an unmeasured confounder and is interpreted as the magnitude of the unmeasured confounder required to draw the observed HR closer to the null (22). The formula HR + √[HR × (HR − 1)] was applied to HRs greater than 1; for HRs less than 1, we took the inverse of the observed HR and then applied the formula.

STATA, version 17 (StataCorp LLC, College Station, Texas), was used for all statistical analyses.

## RESULTS

After exclusions (Figure 1), the study sample consisted of 735,244 women who had their first 2 singleton term births during the period 1967–2020 (Table 1). Spontaneous delivery was registered in 82.3% of first pregnancies and iatrogenic delivery in 17.7%. Women with spontaneous deliveries in the first pregnancy had lower mean maternal age and offspring birth weight, were more frequently smokers, and more often had a low educational level than women with iatrogenic deliveries. On the other hand, women with iatrogenic deliveries had a higher proportion of pregnancy complications, a higher proportion of offspring with congenital anomalies, and more frequently conception by in vitro fertilization. The most common complications among iatrogenic births were preeclampsia, chronic/gestational hypertension, and pregestational/gestational diabetes mellitus.

**Table 1:** Characteristics of First Pregnancies (as Registered in the Medical Birth Registry of Norway) for 735,244 Women Whose First 2 Offspring Were Singleton Term Births, Norway, 1967–2020

	**All Women**		**Type of Labor Onset in First Pregnancy**			
			**Spontaneous Delivery** ^**a**^		**Iatrogenic Delivery** ^**b**^	
**Characteristic**	**No.**	**%**	**No.**	**%**	**No.**	**%**
No. of women	735,244	100	605,419	82.3	129,825	17.7
Maternal age, years^c^	24.7 (4.4)		24.5 (4.4)		25.5 (4.7)	
Offspring birth weight, g^c^	3,514.8 (474.9)		3,505.6 (459.8)		3,557.8 (537.7)	
Maternal education, years^d^						
<11 (low)	129,526	17.7	107,723	17.9	21,803	16.9
≥11 (high)	601,642	82.3	494,450	82.1	107,192	83.1
Maternal birth in a Nordic country^e^	605,684	91.9	498,125	92.1	107,559	90.9
Pregnancy complications						
Pregestational/gestational diabetes mellitus	4,860	0.7	2,470	0.4	2,390	1.8
Chronic/gestational hypertension	15,018	2.0	9,759	1.6	5,259	4.1
Perinatal mortality	3,271	0.4	1,869	0.3	1,402	1.1
Placental abruption	1,826	0.3	1,174	0.2	652	0.5
Preeclampsia	25,800	3.5	11,644	1.9	14,156	10.9
Full-term birth (39–41 weeks’ gestation)	527,206	71.6	452,920	85.9	74,286	14.1
Congenital anomaly in offspring	24,773	3.4	19,185	3.2	5,588	4.3
In vitro fertilization^f^						
No	472,808	98.8	346,670	98.9	81,138	98.2
Yes	5,394	1.2	3,927	1.1	1,468	1.8
Cigarette smoking^g^						
No	172,757	91.5	138,205	91.2	34,552	92.6
Yes	16,131	8.5	13,375	8.8	2,756	7.4

^a^ Women with spontaneous onset of labor during the first pregnancy.

^b^ Women with either induced labor onset or prelabor cesarean delivery during the first pregnancy.

^c^ Values are expressed as mean (standard deviation).

^d^ Information was missing for 4,076 women (0.6%).

^e^ Nordic countries included Norway, Denmark, Finland, Iceland, and Sweden. Information was missing for 76,149 women (10.4%).

^f^ Data on in vitro fertilization were available from 1985 onward (*n* = 478,202).

^g^ Data on smoking were available from 1999 onward (*n* = 237,016). Information was missing for 48,128 women (20.3%).

**Figure 2:**
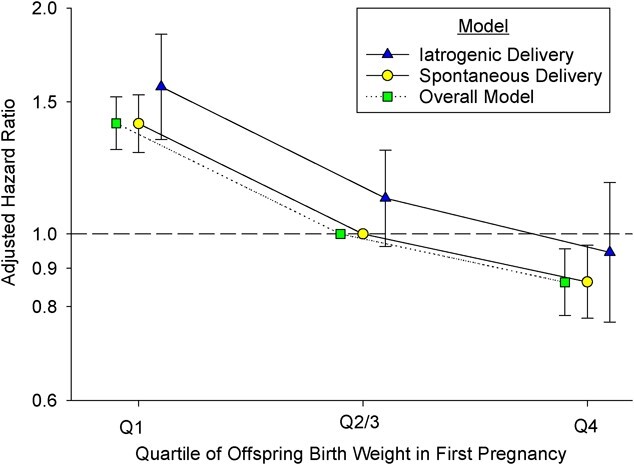
Adjusted hazard ratios for long-term maternal cardiovascular disease mortality by quartile (Q) of offspring birth weight among women whose first 2 singletons were born at term (*n* = 735,244), based on women’s first birth and stratified by onset of labor, Norway, 1967–2020. Iatrogenic deliveries included women with either induced onset of labor or prelabor cesarean delivery during the first pregnancy; spontaneous deliveries included women with spontaneous onset of labor during the first pregnancy. Women with offspring in Q2/3 and spontaneous labor onset during the first pregnancy were the common reference group for the model including spontaneous and iatrogenic deliveries. Women with the first offspring in Q2/3 were the reference group in the overall model. Hazard ratios were adjusted for maternal age at first birth, year of last delivery, maternal education, and pregnancy complications (chronic or gestational hypertension, pregestational or gestational diabetes mellitus, placental abruption, preeclampsia, perinatal loss, congenital malformations, and conception by in vitro fertilization) in the first and/or second pregnancies. Bars, 95% confidence intervals.

**Figure 3:**
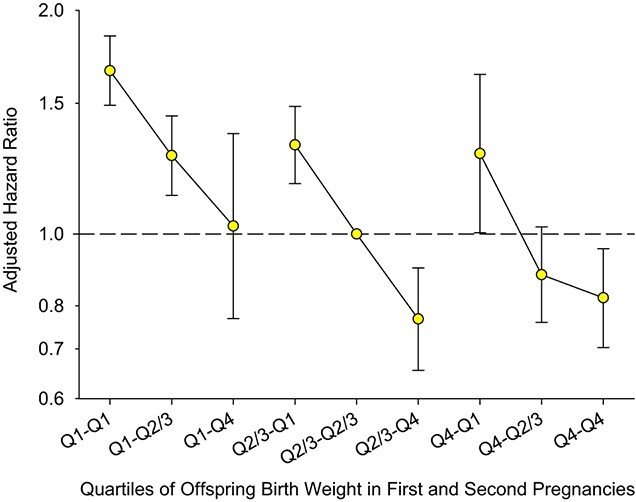
Adjusted hazard ratios for long-term maternal cardiovascular disease mortality by quartile (Q) of offspring birth weight among women whose first 2 singletons were born at term (*n* = 735,244), based on women’s first and second births, Norway, 1967–2020. Hazard ratios were adjusted for maternal age at first birth, year of last delivery, maternal education, and pregnancy complications (chronic or gestational hypertension, pregestational or gestational diabetes mellitus, placental abruption, preeclampsia, perinatal loss, congenital malformations, and conception by in vitro fertilization) in the first and/or second pregnancies. Women whose first 2 offspring were in Q2/3 were the reference group. Bars, 95% confidence intervals.

Among the 735,244 included women, 32,129 died, with 3,037 deaths being from cardiovascular causes. In Figure 2 (Web Table 3), we present data on maternal CVD death based on first offspring quartiles (overall model) and stratified by onset of labor in the first pregnancy. Compared with women whose first offspring was delivered spontaneously with a standardized birth weight in Q2/3, mortality was highest among women whose first offspring’s birth weight was in Q1, ranging from HR = 1.41 (95% CI: 1.28, 1.54) for spontaneous delivery to HR = 1.48 (95% CI: 1.26, 1.74) for iatrogenic delivery. On the other hand, mortality was lowest (HR = 0.86, 95% CI: 0.77, 0.96) among women with spontaneous delivery and a first offspring in Q4. Figure 3 (Web Table 4) presents adjusted HRs for CVD mortality based on information from both the first and second offspring birth weight quartiles. Regardless of first offspring birth weight quartile, there was a decreasing trend in HR estimates if the second offspring was larger. Maternal mortality was highest if both offspring were in Q1 (HR = 1.66, 95% CI: 1.49, 1.85), as compared with women whose first 2 births were in Q2/3. The risk increase associated with a first infant in Q1 was eliminated, however, if the second offspring was in Q4 (HR = 0.99; 95% CI: 0.75, 1.31). For women with a first offspring in Q2/3, the risk of CVD death was higher if the second offspring was in Q1 (HR = 1.33, 95% CI: 1.18, 1.50) but lower if the second offspring was in Q4 (HR = 0.78, 95% CI: 0.67, 0.91). Similarly, for women who started out with an offspring in Q4, the relative mortality risk was highest if the second child was in Q1 (HR = 1.26, 95% CI: 0.99, 1.60) and lowest if the second child was also in Q4 (HR = 0.80, 95% CI: 0.69, 0.93).

**Figure 4:**
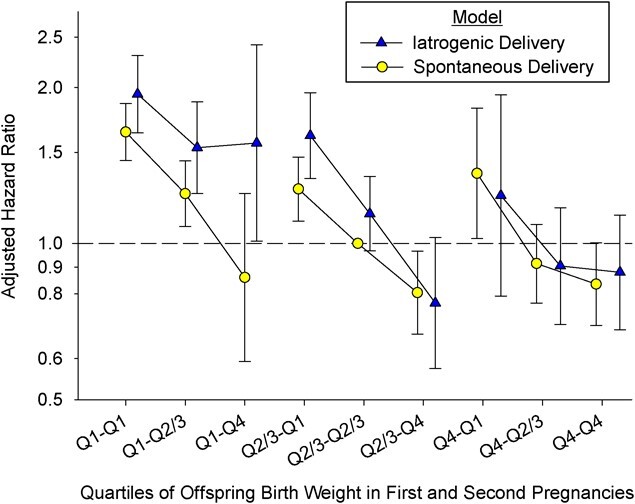
Adjusted hazard ratios for long-term maternal cardiovascular disease mortality by quartile (Q) of offspring birth weight among women whose first 2 singletons were born at term (*n* = 735,244), based on women’s first and second births and stratified by onset of labor, Norway, 1967–2020. Iatrogenic deliveries included women with either induced onset of labor or prelabor cesarean delivery in the first and/or second pregnancy (*n* = 216,283); spontaneous deliveries included women with spontaneous labor onset in both the first and second pregnancies (*n* = 518,961). Women with offspring in Q2/3 and spontaneous onset of labor during both the first pregnancy and the second pregnancy were the reference group. Hazard ratios were adjusted for maternal age at first birth, year of last delivery, maternal education, and pregnancy complications (chronic or gestational hypertension, pregestational or gestational diabetes mellitus, placental abruption, preeclampsia, perinatal loss, congenital malformations, and conception by in vitro fertilization) in the first and/or second pregnancies. Bars, 95% confidence intervals.

A total of 518,961 women (70.6%) had spontaneous deliveries in both pregnancies, while 216,283 women (29.4%) had an iatrogenic delivery in the first and/or second pregnancy. Among women with a spontaneous first delivery, 11.8% had an iatrogenic delivery in the second pregnancy, while 5.6% of the women had an iatrogenic delivery in both pregnancies. Figure 4 (Web Table 5) shows maternal CVD mortality based on information from both the first and the second births, stratified by labor onset. Compared with having 2 births in Q2/3, HR estimates decreased if the second offspring was larger than the first and increased if it was smaller, independent of delivery type (iatrogenic or spontaneous). In most of the quartile groups, women with iatrogenic delivery had higher relative mortality risk than women with spontaneous delivery; however, 95% CIs overlapped. If first births were in Q1, point estimates for women with induced deliveries were higher. The differences were smaller for women with first births in Q2/Q3 and not visible for first births in Q4.

Sensitivity analysis excluding women with pregnancy complications, offspring congenital anomalies, and conception by in vitro fertilization did not change the CVD mortality pattern but attenuated risk (Web Table 6). Similarly, restricting the analysis to women born in Nordic countries who had children with the same partner or to births with a gestational age of 39–41 weeks did not change the mortality pattern. Adjusting for all of these factors, including interpregnancy interval, did not change the mortality patterns.

E-values ranged from 1.11 to 2.71, implying that unmeasured confounding of this extent was required to explain the observed associations. We also observed similar patterns between change in offspring birth weight quartiles from the first pregnancy to the second pregnancy and maternal risk of dying from all causes, circulatory causes, and noncirculatory causes (Web Table 7). Mortality estimates were similar in both hazard models (cause-specific and subdistribution) (Web Table 8). Finally, mortality patterns were similar for women with 3 term births (Web Table 9).

Out of 1,088,309 women, 7.3% (*n* = 79,289) had missing data on either offspring gestational age or birth weight for the first 2 births. These women were younger and had higher proportions of persons with low education, pregnancy complications, and iatrogenic deliveries than the study population (data not shown). For those who gave birth after 1998, women with missing data were also more often smokers.

## DISCUSSION

Including information on both the first and the second infant’s birth weight by gestational age revealed heterogeneity in long-term maternal CVD mortality which was not captured when only using information based on the first offspring. Women whose first infants had similar birth weights differed in their long-term mortality risk depending on their second infants’ birth weights. This was true for both women with spontaneous deliveries and women with iatrogenic deliveries.

In the present study, we found that women with 2 term births in the lowest birth weight quartile (Q1) had up to 66% increased CVD mortality risk compared with women with 2 births in Q2/3. On the other hand, giving birth to a term second offspring in the highest quartile (Q4) was associated with similar or lower long-term maternal mortality, independent of the first offspring’s birth weight quartile. This was unexpected, as fetal growth acceleration is associated with reduced glucose tolerance (6, 23, 24). One plausible explanation could be that the prevalence of diabetes in Norway was generally low in the earlier years of the registry (25), when 75% of the mothers who died from CVD in our study had their first child. Other explanations could be socioeconomic status and behavioral risk factors. Women giving birth to large infants were highly educated and less likely to smoke (during the years when smoking was registered).

Changes in offspring birth weight quartiles from the first birth to the subsequent birth seem to capture heterogeneity in maternal CVD mortality risk and illustrate that moving from one birth weight quartile to another between the first birth and the second adds valuable information with regard to a woman’s future risk of CVD death: Within all birth weight quartiles of first offspring, maternal relative risk of CVD death decreased by increasing second offspring quartile. Moving from higher quartiles of offspring birth weight to lower quartiles in consecutive births was, in most cases, associated with a higher mortality risk than was found for women with both infants in the middle birth weight quartiles. However, moving from a lower birth weight quartile to a higher quartile was only associated with reduced mortality risk when the first offspring was in Q2/Q3 and the second was in Q4, indicating that having a first infant in the lowest birth weight quartile is a relatively stable marker of future mortality risk. This heterogeneity in CVD risk according to change in offspring birth weight quartiles might be masked if only the first infant’s birth weight information is used, as previous studies have done (2–8, 10).

CVD mortality has been found to be higher in iatrogenic deliveries than in spontaneous preterm deliveries (9, 26). The explanation for this is likely to be the higher risk of additional adverse pregnancy complications in women with iatrogenic preterm deliveries which also may be the underlying cause of preterm delivery (26). However, in this study, we found a less clear distinction between spontaneous and iatrogenic term deliveries, which could have been due to a healthier population of women, since we included only term births. For women with a first birth in Q1, however, the risk seemed higher in the iatrogenic group. In general, term complications were more common in the iatrogenic group, which could indicate that pregnancies in this group were more often affected by conditions related to placental dysfunction (27). Preeclampsia, for instance, is a well-known complication associated with women’s long-term CVD mortality (16). However, it is possible that term complications are associated with future CVD mortality risk to a lesser extent than preterm complications, since complications that reach term may be less severe than similar complications with preterm delivery. Severity of complications may also be a factor of importance for future maternal mortality risk—shown, for instance, for preterm preeclampsia, which has a stronger association with future CVD mortality than does term preeclampsia (11). Changes in obstetrical practice have resulted in an increase in the number of women undergoing induction of labor or prelabor cesarean delivery (28), which could influence offspring gestational age and birth weight (12) and may also have influenced our classifications of birth weight quartiles. With the rise in interventions, there has been an increase in the number of women giving birth at early term, which is associated with increased risk of CVD mortality (9). However, excluding these women did not change the pattern of mortality by offspring birth weight quartile.

Strengths of this study include its population-based design, the large sample size, prospectively collected data, and low proportions of missing data. Long-term mortality risk was assessed using information from women’s 2 subsequent births (both live births and stillbirths). We had follow-up for maternal deaths occurring up to 53 years after women’s first birth, median follow-up being 24 years. By using standardized offspring birth weight and parity-specific cutoff values when grouping infants into quartiles, we minimized the possibility of exposure misclassification. The use of observed birth weight–by–gestational age charts in the term population is likely to have been reasonably valid, with little variation and bias (29). The majority of women in Norway continue on to a second pregnancy (11), and restricting our analysis to the first 2 births among women with 2 or more births was likely to limit the influence of selection.

There were some limitations in our study, however, including lack of data on CVD risk factors such as nutritional intake, physical activity, and other environmental factors. Pregnant women were not routinely screened for gestational diabetes before the mid-1980s in Norway (25). Similarly, the validity of data on the onset of labor was low during this first period (14). Our study population included women with the first 2 term births, while excluding those with missing data on birth weight and gestational age. Most of the missing information was accounted for by missing data on gestational age. We therefore used absolute birth weight quartiles to compare CVD mortality patterns among all women and excluding those with missing gestational age data in the first 2 pregnancies. We used 2,500 g as the lower limit of the first quartile to have a “cutoff value” leaning towards preterm births. We found a similar mortality pattern, showing that exclusion of the women with missing gestational age data did not change our result. Data on smoking and BMI were only available for the later years. To account for unmeasured confounding by smoking and BMI, we conducted a sensitivity analysis using E-values, which revealed that a substantial unmeasured confounder with an HR of at least 2.71 would be required to explain the observed HR associated with consecutive births in Q1. Given that not all unmeasured confounders are working in the same direction, the E-value of 2.71 was probably a minimum value of what would be needed for smoking to fully explain our observed association in the Q1-Q1 birth weight category, where smoking was estimated to be most prevalent. Furthermore, in a Swedish cohort study evaluating fetal growth and later maternal CVD, results were not altered after adjustment for smoking and BMI (10). Moreover, we argue that our most robust finding is likely to hold even after adjustment for both BMI and smoking, as the Swedish and Norwegian populations are similar in terms of population characteristics and universal free and accessible health care. Some women may give birth to constitutionally small babies whose small size was not caused by any pathological processes (19). Finally, we expect that these results would apply to other populations with similar population characteristics.

### Health implications

Current guidelines (30) recommend enhanced screening for CVD among women with a history of low offspring birth weight. Given that a majority of women have more than 1 child (84% in Norway) (11), failing to include information on subsequent offsprings’ birth weight may be a missed opportunity for identifying women at high risk of CVD mortality. Moreover, risk factor identification based solely on the first birth may in fact be erroneous. Change in offspring birth weight quartiles could capture heterogeneity in CVD risk, allowing for more precise prediction of mothers’ future risk of CVD death.

### Conclusion

Changes in offspring birth weight quartile from the first pregnancy to the second may offer important information on heterogeneity in women’s future risk of CVD death. Within all birth weight quartiles of first offspring, maternal relative risk of dying from CVD decreased by increasing quartile of the second offspring, with a similar pattern being observed among spontaneous and iatrogenic deliveries. Women with a first offspring in the lowest birth weight quartile seem to have more consistently increased CVD mortality risk and may benefit from intervention aimed at preventing and reducing future risk of CVD. Our findings highlight the importance of including information from women’s subsequent births for identification of high-risk subgroups for specific follow-up.

## Supplementary Material

Web_Material_kwad075
