# Supplementary material for: Birth Weight in Consecutive Pregnancies and Maternal Cardiovascular Disease Mortality Among Spontaneous and Iatrogenic Term Births: A Population-Based Cohort Study
Source: Am J Epidemiol. 2023 May 30;192(8):1326–34. doi: 10.1093/aje/kwad075 (PMC10403302; doi:10.1093/aje/kwad075)
Supplement: Web_Material_kwad075 [file web_material_kwad075.pdf]

WEB MATERIAL

Birth Weight in Consecutive Pregnancies and Long-Term Maternal Cardiovascular Disease Mortality Among Spontaneous and Iatrogenic Term Births: A Population-Based Cohort Study

Yeneabeba Tilahun Sima, Rolv Skjaerven, Liv Grimstvedt Kvalvik, Nils-Halvdan Morken, Kari Klungsøyr, Janne Mannseth, and Linn Marie Sørbye

Yeneabeba Tilahun Sima is a PhD research fellow at the Faculty of Medicine, University of Bergen (Bergen, Norway) and the corresponding author of this paper (Yeneabeba.Sima@uib.no).

Contents:

Web Tables 1–9

Web Table 1. STROBE Statement—Checklist of Items That Should Be Included in Reports of Cohort Studies

	Item No.	Recommendation	Author's Response
**Title and abstract**	1	(*a*) Indicate the study's design with a commonly used term in the title or the abstract	See Title.
		(*b*) Provide in the abstract an informative and balanced summary of what was done and what was found	See Abstract.
**Introduction**			
Background/rationale	2	Explain the scientific background and rationale for the investigation being reported	See Introduction, paragraph 1 and 2.
Objectives	3	State specific objectives, including any prespecified hypotheses	See Introduction, paragraph 3.
**Methods**			
Study design	4	Present key elements of study design early in the paper	See Title and "Data sources" in Material and Methods, paragraph 1.
Setting	5	Describe the setting, locations, and relevant dates, including periods of recruitment, exposure, follow-up, and data collection	See "Data sources" in Material and Methods, paragraph 1.
Participants	6	(*a*) Give the eligibility criteria, and the sources and methods of selection of participants. Describe methods of follow-up	See "Inclusions and definitions" in Material and Methods, paragraph 1 and Figure 1.

		(*b*) For matched studies, give matching criteria and number of exposed and unexposed	
Variables	7	Clearly define all outcomes, exposures, predictors, potential confounders, and effect modifiers. Give diagnostic criteria, if applicable	See "Inclusions and definitions", in Material and Methods, paragraph 2 and 3. Diagnostic criteria see "Inclusions and definitions", paragraph 2.
Data sources/ measurement	8*	For each variable of interest, give sources of data and details of methods of assessment (measurement). Describe comparability of assessment methods if there is more than one group	See "Data sources" and "Inclusions and definitions", in Material and Methods.
Bias	9	Describe any efforts to address potential sources of bias	See "Statistical analyses".
Study size	10	Explain how the study size was arrived at	See "Inclusions and definitions", in Material and Methods, paragraph 1 and Figure 1.
Quantitative variables	11	Explain how quantitative variables were handled in the analyses. If applicable, describe which groupings were chosen and why	See "Inclusions and definitions" in Material and Methods, paragraph 2 and 3. See Table1
Statistical methods	12	(*a*) Describe all statistical methods, including those used to control for confounding	See "Statistical analyses". See S5 Table, S6 Table and S7 Table.
		(*b*) Describe any methods used to examine subgroups and interactions	See "Statistical analyses", paragraph 2.
		(*c*) Explain how missing data were addressed	See "Table 1"
		(*d*) If applicable, explain how loss to follow-up was addressed	
		(*e*) Describe any sensitivity analyses	See "Statistical analyses", paragraph 3.

Results			
Participants	13*	(a) Report numbers of individuals at each stage of study—eg numbers potentially eligible, examined for eligibility, confirmed eligible, included in the study, completing follow-up, and analysed	See "Inclusions and definitions" in Material and Methods, paragraph 1 and Fig1 shows the flow-chart.
		(b) Give reasons for non-participation at each stage	See "Inclusions and definitions" in Material and Methods, paragraph 1, and Figure 1 flow chart.
		(c) Consider use of a flow diagram	See Figure 1.
Descriptive data	14*	(a) Give characteristics of study participants (eg demographic, clinical, social) and information on exposures and potential confounders	See Table1.
		(b) Indicate number of participants with missing data for each variable of interest	See Table1.
		(c) Summarise follow-up time (eg, average and total amount)	See Discussion, paragraph 5.
Outcome data	15*	Report numbers of outcome events or summary measures over time	See Result, paragraph 2.
Main results	16	(*a*) Give unadjusted estimates and, if applicable, confounder-adjusted estimates and their precision (eg, 95% confidence interval). Make clear which confounders were adjusted for and why they were included	See all Figures, Tables and Supplementary Tables
		(*b*) Report category boundaries when continuous variables were categorized	
		(*c*) If relevant, consider translating estimates of relative risk into absolute risk for a meaningful time period	See Supplementary Tables.
Other analyses	17	Report other analyses done—eg analyses of subgroups and interactions, and sensitivity analyses	See all Supplementary files

**Discussion**			
Key results	18	Summarise key results with reference to study objectives	See Discussion, paragraph 1.
Limitations	19	Discuss limitations of the study, taking into account sources of potential bias or imprecision. Discuss both direction and magnitude of any potential bias	See Discussion, paragraph 5 and 6.
Interpretation	20	Give a cautious overall interpretation of results considering objectives, limitations, multiplicity of analyses, results from similar studies, and other relevant evidence	See the different paragraphs of the Discussion.
Generalisability	21	Discuss the generalisability (external validity) of the study results	See Discussion, paragraph 5 and "Health Implications".
**Other information**			
Funding	22	Give the source of funding and the role of the funders for the present study and, if applicable, for the original study on which the present article is based	See information in the "Financial Disclosure" field in the submission form.

STROBE, Strengthening the Reporting of Observational Studies in Epidemiology.

*Give information separately for exposed and unexposed groups.

Note: An Explanation and Elaboration article discusses each checklist item and gives methodological background and published examples of transparent reporting. The STROBE checklist is best used in conjunction with this article (freely available on the Web sites of *PLoS Medicine* at http://www.plosmedicine.org/, *Annals of Internal Medicine* at http://www.annals.org/, and *Epidemiology* at http://www.epidem.com/). Information on the STROBE Initiative is available at http://www.strobe-statement.org.

Web Table 2. Parity specific cutoff points for quartiles of offspring birth weight by gestational age among women with first and second singleton term births during 1967–2020, The Medical Birth Registry of Norway

Gestational Age (weeks)	Mean	Standard Deviation	Q1(≤25th Percentile)	Q2/3 (>25th & ≤75th Percentile)	Q4 (>75th Percentile)
First birth [a]					
37	3058	486	2760	3060	3350
38	3234	454	2950	3230	3520
39	3396	439	3110	3390	3678
40	3528	442	3235	3520	3810
41	3637	455	3330	3630	3930
42	3697	473	3380	3690	4000
43	3664	492	3340	3650	3980
44	3582	486	3260	3570	3900
45	3576	488	3270	3560	3900
46	3578	503	3260	3570	3900
Second birth [b]					
37	3197	505	2880	3180	3500
38	3389	466	3080	3372	3680
39	3546	449	3250	3530	3830
40	3684	451	3380	3670	3970
41	3794	463	3480	3780	4100
42	3842	484	3520	3830	4160
43	3771	508	3430	3760	4100
44	3695	484	3370	3700	4000
45	3700	495	3380	3685	4020
46	3678	516	3335	3680	4010

[a]All women with first singleton term birth, between 1967–2014.

[b]All women with second singleton term birth, between 1968–2020.

Web Table 3. Long-term cardiovascular disease mortality by quartiles (Q) of offspring birth weight by gestational age, based on women's first birth, and stratified by onset of labor: Women with first two singleton births at term, Norway, 1967–2020 (*n* = 735,244)

Quartile of Birth Weight by Gestational Age	Model 1				Model 2							
	Overall Model				Spontaneous Deliveries [a]				Iatrogenic Deliveries [b]			
	n	N	n/N	aHR[c] (95% CI)	n	N	n/N	aHR[c] (95% CI)	n	N	n/N	aHR[c] (95% CI)
1st birth												
Q1	1,118	179,230	0.62	1.41 (1.30-1.52)	939	147,182	0.64	1.41 (1.28-1.54)	179	32,048	0.56	1.48 (1.26-1.74)
Q2/3	1,389	371,517	0.37	1.00 (reference)[d]	1,172	310,690	0.38	1.00 (reference)[e]	217	60,827	0.36	1.07 (0.92-1.23)
Q4	530	184,497	0.29	0.84 (0.77-0.94)	438	147,547	0.30	0.86 (0.77-0.96)	92	36,950	0.25	0.86 (0.70-1.07)

[a] Women with spontaneous labor onset during first pregnancy.

[b] Women with either induced onset of labor or pre-labor caesarean delivery during first pregnancy.

[c] Adjusted for maternal age at first birth, year of last delivery, maternal education pregnancy complications (chronic- or gestational hypertension, pregestational- or gestational diabetes mellitus, placental abruption, preeclampsia, perinatal loss, offspring with congenital malformations and women who conceived by In vitro fertilization) in first and/ second pregnancies

[d] Women with first offspring in Q2/3.

[e] Women with offspring in Q2/3 and spontaneous onset of labor during first pregnancy, were the common reference group for the model including spontaneous and iatrogenic births.

Web Table 4. Long-term maternal cardiovascular mortality by quartiles (Q) of offspring birth weight by gestational age, based on woman's first and second birth: Women whose first two singleton births at term, Norway, 1967–2020 (*n* = 735,244)

Quartile of Birth Weight by Gestational Age		n	N	n/N	aHR[a] (95% CI)
1st birth	2nd birth				
Q1	Q1	648	86,259	0.75	1.66 (1.49-1.85)
Q1	Q2/3	417	79,729	0.52	1.31 (1.13-1.48)
Q1	Q4	53	13,242	0.40	0.99 (0.75-1.31)
Q2/3	Q1	453	82,832	0.55	1.33 (1.18-1.50)
Q2/3	Q2/3	732	208,993	0.35	1.00 (reference)
Q2/3	Q4	204	79,692	0.26	0.78 (0.67-0.91)
Q4	Q1	72	13,979	0.52	1.26 (0.99-1.60)
Q4	Q2/3	240	80,331	0.30	0.89 (0.77-1.03)
Q4	Q4	218	90,187	0.24	0.80 (0.69-0.93)

[a] Hazard ratio with 95% confidence interval, adjusted for maternal age at first birth, year of last delivery, maternal education and pregnancy complications (chronic- or gestational hypertension, pregestational- or gestational diabetes mellitus, placental abruption, preeclampsia, perinatal loss, offspring with congenital malformations and women who conceived by In vitro fertilization) in first and/ second pregnancies.

Web Table 5. Long-term maternal cardiovascular disease mortality by quartiles (Q) of offspring birth weight by gestational age, based on women's first and second birth: Women with first two singleton births at term, Norway, 1967–2020 (*n* = 735,244)

**Quartile of Birth Weight by Gestational Age**		**Spontaneous Deliveries [a]**				**Iatrogenic Deliveries [b]**			
		**n**	**N**	**n/N**	**aHR[c] (95% CI)**	**n**	**N**	**n/N**	**aHR[c] (95% CI)**
1st birth	2nd birth								
Q1	Q1	466	61,404	0.76	1.66 (1.46-1.88)	182	24,855	0.73	1.86 (1.56-2.20)
Q1	Q2/3	297	57,355	0.52	1.30 (1.13-1.50)	120	22,374	0.54	1.49 (1.22-1.81)
Q1	Q4	29	8,859	0.33	0.83 (0.57-1.20)	24	4,383	0.55	1.46 (0.96-2.22)
Q2/3	Q1	314	59,489	0.53	1.30 (1.13-1.50)	139	23,343	0.60	1.55 (1.28-1.87)
Q2/3	Q2/3	532	153,358	0.35	1.00 (reference)	200	55,635	0.36	1.11 (0.94-1.31)
Q2/3	Q4	154	56,155	0.27	0.84 (0.70-1.00)	50	23,537	0.21	0.71 (0.53-0.95)
Q4	Q1	51	9,238	0.55	1.35 (1.01-1.79)	21	4,741	0.44	1.19 (0.77-1.84)
Q4	Q2/3	175	54,809	0.32	0.94 (0.79-1.11)	65	25,522	0.25	0.86 (0.66-1.11)
Q4	Q4	150	58,294	0.26	0.83 (0.69-1.00)	68	31,893	0.21	0.80 (0.62-1.04)

[a] Women with spontaneous labor onset in first and second pregnancies (n = 518,961).

[b] Women with either induced onset of labor or pre-labor caesarean delivery, in first and/ second pregnancies (n = 216,283).

[c] Hazard ratio with 95% confidence interval, adjusted for maternal age at first birth, year of last delivery, maternal education and pregnancy complications (chronic- or gestational hypertension, pregestational- or gestational diabetes mellitus, placental abruption, preeclampsia, perinatal loss, offspring with congenital malformations and women who conceived by In vitro fertilization) in first and/ second pregnancies.

Web Table 6. Adjusted hazard ratios (HRs) for long-term maternal cardiovascular disease mortality by quartiles(Q) of offspring birth weight by gestational age, based on woman's first and second birth: Women with first two singleton births at term, Norway, 1967–2020 (*n* = 735,244)

Quartile of Birth Weight by Gestational Age		Model [a]	Model [b]	Model [c]	Model [d]	Model [e]	E-Value [f]	
		aHR[a] (95% CI)	aHR[a] (95% CI)	aHR[a] (95% CI)	aHR[a] (95% CI)	aHR[a] (95% CI)	For aHR	For CI
1st birth	2nd birth							
Q1	Q1	1.80 (1.61-1.99)	1.66 (1.49-1.85)	1.62 (1.45-1.82)	1.52 (1.31-1.77)	1.67 (1.46-1.91)	2.71	2.24
Q1	Q2/3	1.37 (1.22-1.55)	1.31 (1.16-1.48)	1.30 (1.14-1.49)	1.25 (1.05-1.48)	1.34 (1.16-1.56)	1.95	1.49
Q1	Q4	1.08 (0.82-1.42)	0.99 (0.75-1.31)	0.98 (0.70-1.35)	1.08 (0.73-1.60)	1.24 (0.90-1.71)	1.11	1.00
Q2/3	Q1	1.38 (1.23-1.55)	1.33 (1.18-1.50)	1.29 (1.13-1.47)	1.27 (1.08-1.50)	1.34 (1.16-1.56)	1.99	1.54
Q2/3	Q2/3	1.00 (reference)	1.00 (reference)	1.00 (reference)	1.00 (reference)	1.00 (reference)	-	-
Q2/3	Q4	0.78 (0.67-0.91)	0.78 (0.67-0.91)	0.80 (0.67-0.94)	0.78 (0.62-0.96)	0.86 (0.71-1.03)	1.88	1.00
Q4	Q1	1.28 (1.01-1.63)	1.26 (0.99-1.60)	1.28 (0.98-1.67)	1.26 (0.89-1.78)	1.30 (0.96-1.76)	1.83	1.00
Q4	Q2/3	0.88 (0.76-1.02)	0.89 (0.77-1.03)	0.87 (0.74-1.02)	0.82 (0.67-1.01)	0.92 (0.77-1.10)	1.49	1.00
Q4	Q4	0.80 (0.68-0.93)	0.80 (0.69-0.93)	0.77 (0.65-0.92)	0.82 (0.66-1.01)	0.75 (0.62-0.91)	1.81	1.37

[a] All first and second term births, unadjusted.

[b] All first and second term births, adjusted for maternal age at first delivery, year of last delivery, maternal education and pregnancy complications (chronic- or gestational hypertension, pregestational- or gestational diabetes mellitus, placental abruption, preeclampsia, perinatal loss, offspring with congenital malformations and women who conceived by In vitro fertilization) in first and/ second pregnancies.

[c] Excluding women with any of the listed pregnancy complications during any of their two first pregnancies. Models adjusted for maternal age at first delivery, year of last delivery, maternal education.

[d] Restricted to births within 39-41 gestational weeks, in both pregnancies. Model adjusted for maternal age at first delivery, year of last delivery, maternal education and above listed pregnancy complications in first and/ second pregnancies.

[e] Restricted to women born in the Nordic countries with offspring from the same father. Model adjusted for maternal age at first delivery, year of last delivery, maternal education, inter-pregnancy-interval and above listed pregnancy complication in first and/ second pregnancies.

[f] E-value is the minimum strength of association that an unmeasured confounder would need to have with both the exposure and outcome, conditional on the measured covariates, to fully explain away a specific exposure–outcome association. E-values for HR and CI was calculated, according to the formula introduced by Tyler J. VanderWeele and Peng Ding (2017), based on model[b].

Web Table 7. Adjusted hazard ratios (HRs) for maternal mortality from all-causes, circulatory, and non-circulatory causes by quartiles (Q) of offspring birth weight by gestational age, based on woman's first and second birth: Women with first two singleton births at term, Norway, 1967–2020 (*n* = 735,244)

Quartile of Birth Weight by Gestational Age		All-Cause Mortality aHR[a] (95% CI)	Circulatory Disease Mortality aHR[a] (95% CI)	Non-Circulatory Disease Mortality aHR[a] (95% CI)
1st birth	2nd birth			
Q1	Q1	1.30 (1.26-1.35)	1.55 (1.43-1.68)	1.24 (1.19-1.30)
Q1	Q2/3	1.11 (1.06-1.16)	1.23 (1.13-1.35)	1.08 (1.03-1.13)
Q1	Q4	0.98 (0.89-1.07)	1.00 (0.81-1.24)	0.97 (0.87-1.08)
Q2/3	Q1	1.20 (1.16-1.25)	1.35 (1.24-1.48)	1.17 (1.11-1.22)
Q2/3	Q2/3	1.00 (reference)	1.00 (reference)	1.00 (reference)
Q2/3	Q4	0.92 (0.88-0.97)	0.88 (0.78-0.98)	0.93 (0.88-0.98)
Q4	Q1	1.15 (1.05-1.25)	1.17 (0.97-1.41)	1.14 (1.04-1.26)
Q4	Q2/3	0.94 (0.90-0.99)	0.96 (0.86-1.06)	0.94 (0.89-0.99)
Q4	Q4	0.90 (0.86-0.95)	0.90 (0.80-1.00)	0.90 (0.86-0.95)

[a] Adjusted for maternal age at first delivery, year of last delivery, maternal education and pregnancy complications (chronic- or gestational hypertension, pregestational- or gestational diabetes mellitus, placental abruption, preeclampsia, perinatal loss, offspring with congenital malformations and women who conceived by In vitro fertilization) in in first and/ second pregnancies.

Web Table 8. Adjusted hazard ratios (HRs) for long-term maternal cardiovascular disease mortality by quartiles (Q) of offspring birth weight by gestational age from cause-specific and sub distribution hazard models, based on woman's first and second birth: Women with first two singleton births at term, Norway, 1967–2020 (*n* = 735,244)

Quartile of Birth Weight by Gestational Age		Cause-Specific Hazard Model aHR[a] (95% CI)	Subdistribution Hazard Model aHR[a] (95% CI)
1st birth	2nd birth		
Q1	Q1	1.66 (1.49-1.85)	1.64 (1.48-1.83)
Q1	Q2/3	1.31 (1.13-1.48)	1.31 (1.13-1.48)
Q1	Q4	0.99 (0.75-1.31)	0.99 (0.75-1.32)
Q2/3	Q1	1.33 (1.18-1.50)	1.33 (1.18-1.49)
Q2/3	Q2/3	1.00 (reference)	1.00 (reference)
Q2/3	Q4	0.78 (0.67-0.91)	0.78 (0.67-0.91)
Q4	Q1	1.26 (0.99-1.60)	1.26 (0.99-1.60)
Q4	Q2/3	0.89 (0.77-1.03)	0.89 (0.77-1.03)
Q4	Q4	0.80 (0.69-0.93)	0.80 (0.69-0.93)

[a] Adjusted for maternal age at first delivery, year of last delivery, maternal education and pregnancy complications (chronic- or gestational hypertension, pregestational- or gestational diabetes mellitus, placental abruption, preeclampsia, perinatal loss, offspring with congenital malformations and women who conceived by In vitro fertilization) in in first and/ second pregnancies

Web Table 9. Adjusted hazard ratios (aHRs) for long-term maternal cardiovascular disease mortality by quartiles (Q) of offspring birth weight by gestational age, based on first and third birth: Women with first three singleton births at term, Norway, 1967–2020 (*n* = 268,377)

Quartile of Birth Weight by Gestational Age		Model [a]	Model [b]	Model [c]	
		aHR[a] (95% CI)	aHR[a] (95% CI)	Spontaneous Deliveries [d] aHR[a] (95% CI)	Iatrogenic Deliveries [e] aHR[a] (95% CI)
1st birth	3rd birth				
Q1	Q1	1.81 (1.51-2.16)	1.85 (1.51-2.26)	1.88 (1.50-2.36)	1.92 (1.46-2.52)
Q1	Q2/3	1.19 (0.97-1.47)	1.14 (0.90-1.45)	1.20 (0.92-1.57)	1.34 (0.98-1.84)
Q1	Q4	0.89 (0.55-1.41)	0.90 (0.51-1.57)	0.97 (0.54-1.75)	0.87 (0.41-1.86)
Q2/3	Q1	1.36 (1.11-1.66)	1.29 (1.03-1.62)	1.24 (0.95-1.61)	1.77 (1.33-2.35)
Q2/3	Q2/3	1.00 (reference)	1.00 (reference)	1.00 (reference)	1.14 (0.88-1.47)
Q2/3	Q4	0.86 (0.67-1.11)	0.80 (0.60-1.07)	0.78 (0.56-1.10)	1.11 (0.77-1.59)
Q4	Q1	1.42 (0.98-2.06)	1.32 (0.85-2.05)	1.60 (0.99-2.57)	1.35 (0.75-2.43)
Q4	Q2/3	0.86 (0.67-1.11)	0.85 (0.64-1.12)	0.87 (0.63-1.21)	0.95 (0.65-1.39)
Q4	Q4	0.71 (0.53-0.93)	0.75 (0.55-1.01)	0.82 (0.58-1.15)	0.63 (0.40-0.99)

[a] All first three term births, adjusted for maternal age at first delivery, year of last delivery, maternal education and pregnancy complications (chronic- or gestational hypertension, pregestational- or gestational diabetes mellitus, placental abruption, preeclampsia, perinatal loss, offspring with congenital malformations and women who conceived by In vitro fertilization) in any of the first three pregnancies.

[b] Excluding women with any of the above listed complications during any of their first three pregnancies. Model adjusted for maternal age at first delivery, year of last delivery, maternal education.

[c] All first three term births, stratified by onset of labor. Model adjusted for maternal age at first delivery, year of last delivery, maternal education and above listed pregnancy complications in any of the first three pregnancies.

[d] Women with spontaneous labor onset in first, second and third pregnancies.

[e] Women with either induced onset of labor or pre-labor caesarean delivery, in any of their first three pregnancies.
